# Microstructure Formation and Fracturing Characteristics of Grey Cast Iron Repaired Using Laser

**DOI:** 10.1155/2014/541569

**Published:** 2014-06-16

**Authors:** Peng Yi, Pengyun Xu, Changfeng Fan, Guanghui Yang, Dan Liu, Yongjun Shi

**Affiliations:** College of Mechanical and Electronic Engineering, China University of Petroleum, Qingdao 266580, China

## Abstract

The repairing technology based on laser rapid fusion is becoming an important tool for fixing grey cast iron equipment efficiently. A laser repairing protocol was developed using Fe-based alloy powders as material. The microstructure and fracturing feature of the repaired zone (RZ) were analyzed. The results showed that regionally organized RZ with good density and reliable metallurgical bond can be achieved by laser repairing. At the bottom of RZ, dendrites existed in similar direction and extended to the secondary RZ, making the grains grow extensively with inheritance with isometric grains closer to the surface substrate. The strength of the grey cast iron base material was maintained by laser repairing. The base material and RZ were combined with robust strength and fracture resistance. The prevention and deflection of cracking process were analyzed using a cracking process model and showed that the overall crack toughness of the materials increased.

## 1. Introduction

Large quantities of cast iron equipment are widely applied in the industry, such as power supply, and manufacturing. Noticeable cracks are easily initiated at the surface of cast iron equipment because of the working condition of overload and fatigue. Without timely repair, cracks would result in the failure or even equipment breakdown, which leads to enormous economic losses. However, because of poor welding characteristics of cast iron, conventional approaches that have large heat inputs, long repairing cycles, and bad process controllability cannot satisfy the requirements of repairing technology improvement [1–3]. In recent years, repairing technology based on laser fusion has become an effective method for surface repairing. Laser-fusion repairing technology has highly concentrated heat input, small deformation of heat affected zone and substrate, and excellent process controllability and flexibility, which is significant in cast iron repairing.

Various researches have been done in terms of laser repairing. Dong investigated problems on the repairing process of complex and free curved surface and studied the repairing paths of three-dimensional laser remanufacturing [4]. The algorithm of autogeneration repairing paths was proposed based on equidistant parallel section method. Ma and his coworkers repaired a cold mold using laser repairing, which demonstrated good metallurgical bonding between the repaired layer and the substrate [5]. The wear resistance of the iron-based repaired layer was greatly improved. In a study by Wang et al., titanium alloy cups used in aeroengine were successfully repaired using laser without any pinhole, crack, or other defects [6]. In addition, the feasibility to repair structural member of titanium alloys by pulsed laser cladding was proven. Laser repairing of some deteriorated engineering components, such as sintered tool and turbine blades, was also investigated [7, 8]. All previously conducted research achieved repaired layer with good bonding and usable recovery of the repaired components. Although laser repairing of several equipment has established a strong foundation for industrial application, research about laser repairing of cast iron components remains limited.

Some significant studies have been performed on improving surface properties of cast iron by laser surface modification. Laser hardening of grey cast iron was performed by Sridhar et al. [9]. In his experiments, different microstructures of treated surface were derived with various scanning speed. Microhardness and wear resistance of the hardened layer increased significantly compared with base metal. The cracking characteristics of laser cladding with grey cast iron were studied by Luan et al. and some schemes to suppress cracks were developed [10]. Grey cast iron was processed through laser cladding with Co-based alloy by Ocelík et al., and the experiments demonstrated that the tailoring process parameters can reduce the defect rate and elevate the mechanical properties [11]. Tong and Zhou fabricated a variety of unsmooth biomimetic units on grey cast iron by laser cladding and acquired excellent thermal fatigue resistance and wear resistance [12–14]. These works aim at surface modification of cast iron and provide good theoretical foundation and new methods for laser repairing applications. Therefore, the use of laser in repairing cast iron equipment is feasible and necessary.

The heat responses of grey cast iron substrate are the foundation in laser repairing process. The transient and static response rules of substrate can be revealed by theoretical analysis and numerical methods [15–17]. However, the practical achievement and optimization of laser repairing process also need the determination of material transition characteristics. Consequently, the microstructure characteristics and transition rules are initially analyzed by laser repairing experiments. After laser repairing process, bending tests of the repaired samples are performed to investigate the status and strength of the metallurgical bonding between the RZ and the substrate. The effect of the fracture mechanism under concentrated stress is studied by observation of fracture morphology. Moreover, the cracking procedure and affecting rules of bonding strength in RZs are discussed.

## 2. Laser Repairing Experiments and Materials 

**Figure 1:**
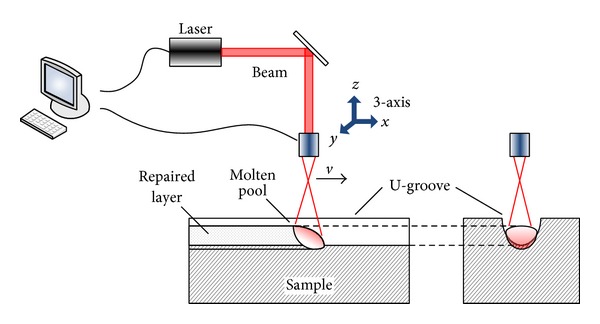
Diagram of a laser repairing system.

**Figure 2:**
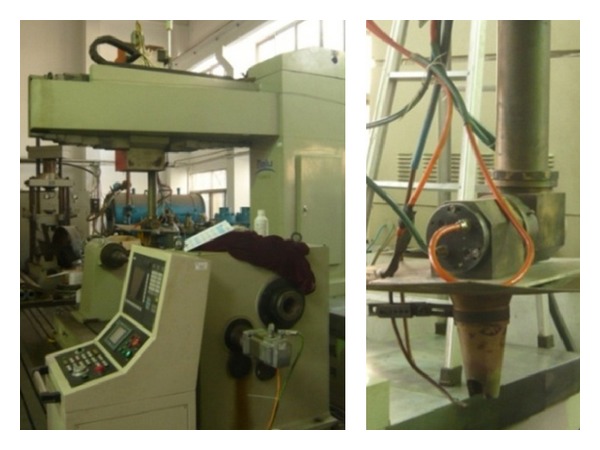
Laser repairing equipment.

A diagram of laser repairing experiment system is demonstrated in Figure 1. The impaired substrate surface was removed with a small amount of material to make a U-groove, and then multilayer repairing was conducted to recover the surface morphology and usability. Grey cast iron was processed by DL-HL-T5000 CO_2_ laser (laser wavelength 10.6 *μ*m, maximum output power 5 kW) and DL-LPM-IV multifunction NC machine, as shown in Figure 2.

In the repairing process, two layers were repaired along the same laser scanning route and direction. The optional laser process parameters are exhibited in Table 1. To melt the two sides of the groove, laser spot radius should be set with slightly larger width than that of the U-groove. In addition, the laser power of the second layer remains constant because the first layer can preheat the second layer, and changing the laser power is time consuming. The scanning speed and laser spot radius during the second layer repairing are enlarged properly to balance the heat input.

**Figure 3:**
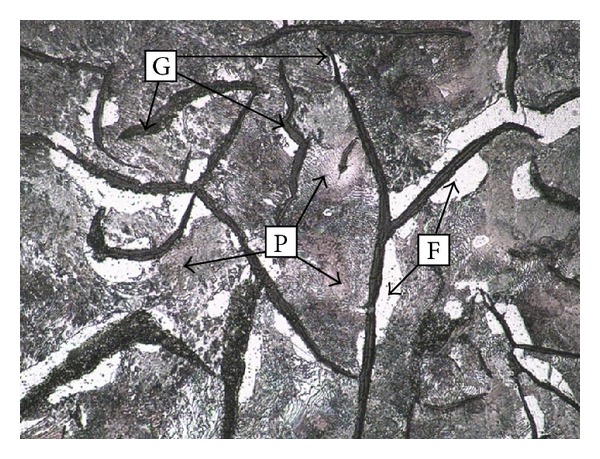
Microstructure of HT250 substrate.

Grey cast iron (HT250) is selected as the pending repaired substrate material. The material is made of iron-based high-carbon multielement alloy and its chemical compositions are listed in Table 2. The carbon existence form of HT250 is graphite grain and Fe-C multielement compounds. The cross-section is corroded by 4% Nital and the microstructure is illustrated in Figure 3. The HT250 microstructure consists of ferrite, pearlite, flake graphite, and a small amount of phosphide eutectic. Sample dimension was about 50 mm × 40 mm × 8 mm. Prior to repair of the grey cast iron surface, the crack was removed. To mimic the operation of crack removal, U-grooves with 3 mm to 4 mm thickness and 4 mm width were fabricated on the sample surface. The samples were ground, polished, and cleaned using absolute alcohol and acetone and then parched.

Alloy powder was preplaced on the U-groove during the laser repairing experiment. The powder and its adjacent substrate material were melted simultaneously when affected by laser radiation. Considering the poor welding characteristics of the grey cast iron, the repair material should have excellent wettability with the substrate and the thermophysical parameters should be well matched. Fe-based self-fluxing powder was selected as the filling material because of the similar composition with grey cast iron and its low cost. The chemical composition of the Fe-based powder with powder size 150 *μ*m to 250 *μ*m and density 2.8 g/cm^3^ to 4.8 g/cm^3^ is exhibited in Table 3.

## 3. Microstructure Characteristics of Fusion Zone

The repaired samples were cooled at room temperature and cut along the vertical direction of the laser repairing path using a DK7725 wire electric discharge machine. The section planes were polished and corroded with 4% Nital. The microstructure of transverse sections under different heat effect treatments was observed and analyzed using HITACHI S-4800 scanning electron microscope (SEM) and MBA-1000 optical microscope (OM), respectively. For convenience of description, the cross-section of the sample was divided into three zones, namely, heat affected zone (HAZ), bond zone (BZ), and repaired zone (RZ).

### 3.1. Heat Affected Zone

**Figure 4:**
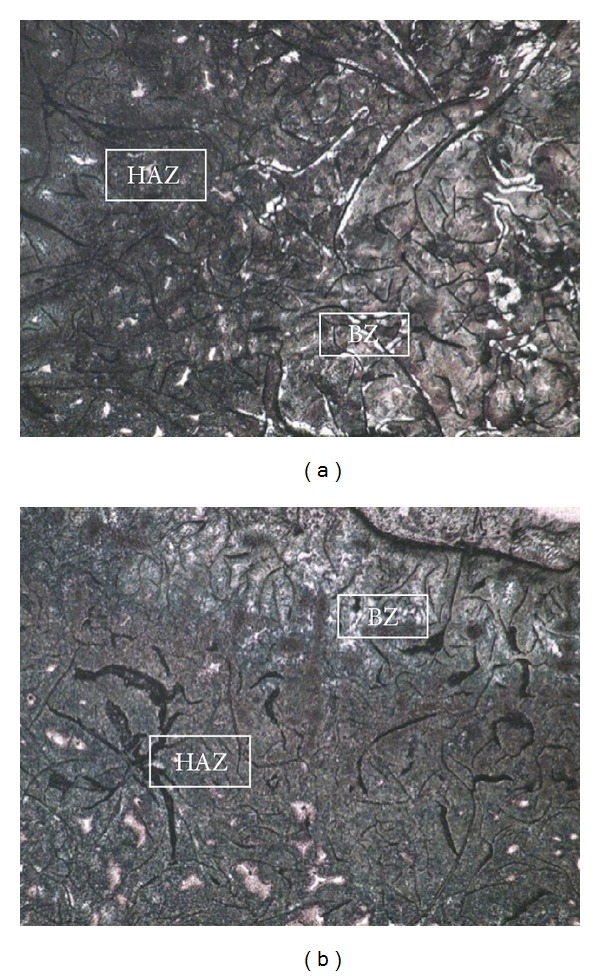
Metallographic structure of HAZ × 200: (a) Fe314 and (b) 316L.

HAZ is located between the BZ and the substrate. The heating temperature of HAZ is between hypoeutectic and eutectoid temperature, and the microstructure only has solid-state phase transition during the repairing process.  Figure 4 illustrates the HAZ microstructure under OM. As there is no generation of molten pool in HAZ, this area has little composition attenuation and infiltration with the bond zone. The microstructure morphology of HAZ in the two sets remains the same. Compared with the bond zone and the RZ, the temperature gradient of HAZ is small and the microstructure that contains much unmelted graphite has a mild transition. The primitive microstructure at the bottom of HAZ consists of abundant pearlite and minute quantity of ferrite, whereas the composition of the microstructure at the top of HAZ is rich in ferrite with some needle-like or cryptocrystalline martensite and blocky cementite at eutectoid temperature.

According to the Fe-C phase diagram and bidirectional transition characteristics of the microstructure, the primitive pearlite in substrate begins to decompose into ferrite and cementite as temperature exceeds the eutectoid point, whereas pearlite transforms into austenite. As temperature continuously increases, the primitive ferrite is converted into austenite with some carbon element decomposing into austenite. The transition of pearlite and ferrite is not complete because of the rapid movement of the molten pool. Therefore, HAZ has unbalanced microstructure including unstable austenite, pearlite, ferrite, and graphite. The temperature declines below the eutectoid point with the passage of molten pool. Austenite begins its transition into ferrite and expands relying on graphite. Some austenite cannot be converted completely and turns into residual austenite because of the rapid cooling rate. After exsolution from austenite, the carbon atom is inactive in solid phase and hardly turns into secondary graphite. Therefore, carbon atom combines with neighboring iron atom into secondary high-carbon cementite. As the temperature decreases to room temperature, a complicated microstructure composed of ferrite, martensite, residual austenite, cementite, and graphite is generated, as illustrated in Figure 4.

### 3.2. Bond Zone

**Figure 5:**
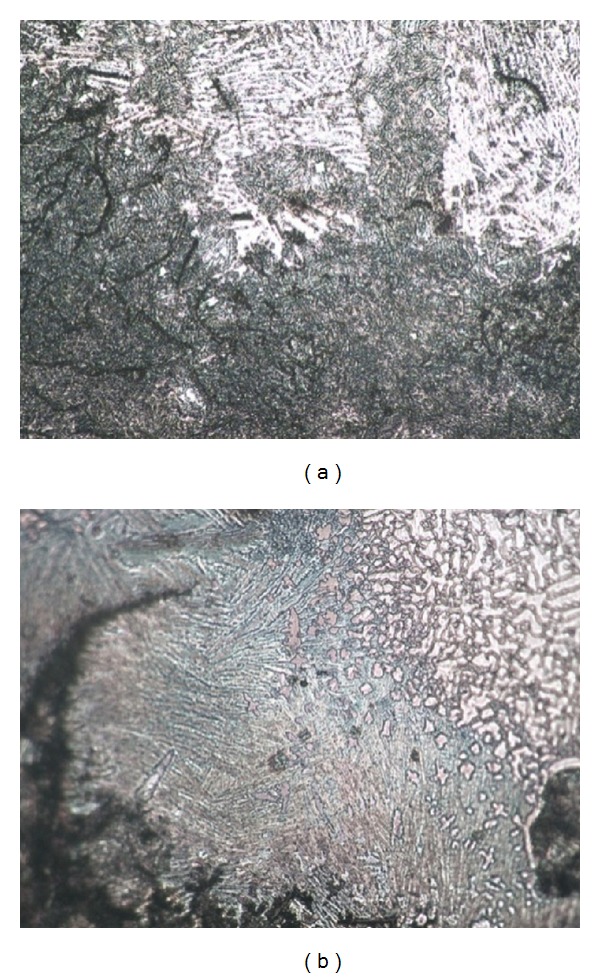
Morphologies of BZ × 200: (a) Fe314, (b) 316L.

The BZ is located between HAZ and RZ. The peak temperature at this area can reach above the eutectic point and belongs to micromelting zone at molten pool brink. The microstructure of BZ had obvious transition, as BZ composition had attenuated and infiltrated under the direct effect of molten pool. The supercooling degree of BZ was higher than those of the other areas. In addition, BZ is under the coactions of rapid melting, solidification, and material attenuation. Therefore, a complicated microstructure is created as demonstrated in Figure 5. The composition phases contain ledeburite, needle-like martensite, ferrite, dendritic austenite, and blocky cementite. The complex phases are caused by the existence of carbon. Copious amounts of graphite are dissolved within the BZ and the carbon element cannot be precipitated into graphite because of supercooling. Consequently, carbon element is dissolved into *α*-Fe and *γ*-Fe and generated ferrite and austenite, respectively. Some carbon element has combined reaction with Fe to generate cementite. High cooling rate changes austenite into eutectic ledeburite with cementite. A small number of austenite transform into pearlite and have eutectic reaction with cementite to produce secondary ledeburite. Some unmelted residual graphite is also present. Hence, a complicated multiphase microstructure is generated in BZ.

Austenite in Figure 5(a) keeps high-carbon content during the rapid cooling process. Ni and *γ*-Fe can produce infinite solid solution, which improves the stability of austenite. However, the hard mechanical mixture, which includes high-carbon martensite and ledeburite, is also generated, because of severe graphite dissolution. The microstructure of 316L in Figure 5(b) is relatively uniform, containing tiny martensite surrounding graphite and some ledeburite, austenite, and cementite at the grain boundaries. It can be seen from the composition of BZ that the microstructure of Fe314 is the mixture of high-carbon martensite and ledeburite, which is more similar with HAZ. However, the microstructure of 316L is mainly tiny needle-like martensite, which is much different from HAZ. Therefore the transitional and bonding function of the Fe314 BZ is much better and obvious than 316L BZ.

**Figure 6:**
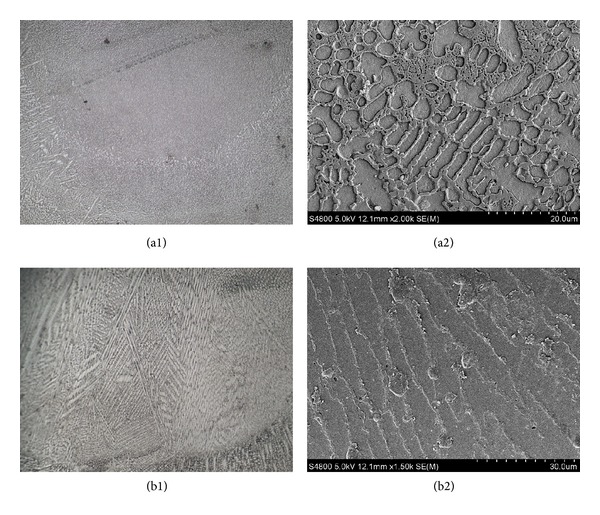
Morphologies of RZ: (a1) Fe314: OM × 100, (a2) Fe314: SEM × 1500, (b1) 316L: OM × 100, and (b2) 316L: SEM × 1500.

With the growth of columnar grain and rapid cooling of molten pool in RZ, the dendrite trunk inherits the growth tendency of the columnar grain in BZ and has obvious extensional growth characteristics [18]. Figures 6(a1) and 6(b1) demonstrate the coarsening of dendrite. However, in the middle of RZ, where increasing degree of supercooling and nucleation rate is observed, the formed columnar grain ceases to develop. Much more new grains begin to accumulate and a fine dendritic microstructure is generated. Grain size and growing direction present a region as a result of nonuniform solute and temperature effect.

Different materials have different grain types. As exhibited in Figure 6(a1), the bonding boundary between RZ and BZ is composed of mainly planar and columnar grains, caused by nucleation, rapid growth, and mutual extrusion. Grains have little branches and turn into isometric grains after transitory growth, as shown in Figure 6(a2). The whole RZ is basically occupied by isometric grain groups, and the coarsening bonding layer only exists at the middle. This observation is mainly produced upon inherited growth of grains, because of microstructure remelts and grain production during second layer repairing.

Comparative microstructure of 316L RZ is a typical austenite stainless steel cellular grain, which is more compact than that of Fe314, as illustrated in Figures 6(b1) and 6(b2). The austenite stainless steel microstructure has large carbon concentration gradient with substrate, which results in obvious microstructure hierarchy in the solidification and phase transition period of BZ. Therefore, the metallurgical bonding strength of 316L is not as strong as Fe314. The formed grains in the first layer can be the nucleation sites of grains in the second layer, because the composition of the two repaired layers is similar. Thus, liquid metal in the molten pool can keep growing on the foundation of the grains within the former RZ, which presents extensively growing characteristics of inheritance [19].

Both sets of the repaired samples have dense and compact repaired microstructure. As one of the strengthening methods, microstructure refinement can improve the hardness and strength of the material, as well as the plasticity and toughness. Thus, RZ can have higher crack resistance than grey cast iron substrate. In addition, martensite transition in the RZ can lead to volume inflation, which produces residual stress, and modify stress status of material surface.

## 4. Fracturing Characteristics Analysis of Repaired Samples 

The RZ and substrate have sufficient metallurgical bonding in laser repairing experiment. Although the matching Fe-based alloy powder is selected as the repairing material, the microstructure and composition in different zones have large differences. The unmelted microstructure phase transition in the heat affected zone is observed, which affected the material properties. Thus, investigating the repaired strength is necessary. Considering the lathy shape of RZ and the brittleness of grey cast iron, bending test is considered to be an appropriate test approach. In the bending test, normal stress on sample surface is large and cracks are sensitive. The test is commonly used to inspect fusion layer properties and characterize bonding strength between fusion layer and substrate [20].

### 4.1. Design of Three-Point Bending Test

**Figure 7:**
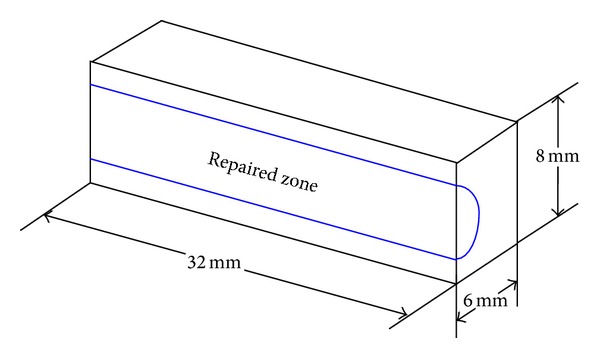
Dimensions of bending test sample.

To compare with the HT250 substrate without laser treatment, two sets of repaired samples, which have the same repairing process and parameters, were selected for bending test. To fully demonstrate the effect of RZ, the samples of the entire RZ should occupy a certain percentage of the whole volume. The test sample was fabricated using wire electric discharge machine, as exhibited in Figure 7.

**Figure 8:**
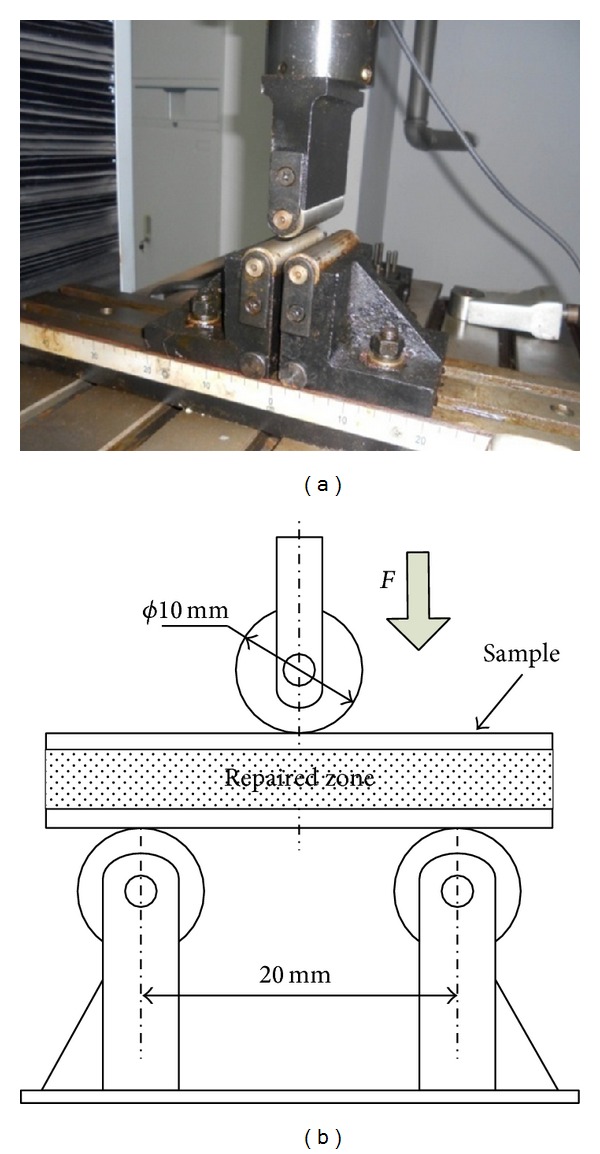
Three-point bending test: (a) testing machine, (b) loading sketch.

The WDW-100D testing machine was used in the bending test. The maximum loading force was 100 kN, with an accuracy grade of 0.5.  Figure 8 schematically depicts the test equipment and the sketch of the sample position.

The span of the two supporting points is 20 mm and the surface of RZ is toward the outside direction. The diameter of top indenter is 10 mm. The substrate and repaired samples are placed on the bottom indenter of the testing machine and suffer the steady load from top indenter. The load and displacement data of different samples in fracturing process are extracted and drawn into the load-displacement curves. The fracture appearances of different samples are observed by S-4800 cold field emission SEM.

### 4.2. Fracture Appearances Analysis

**Figure 9:**
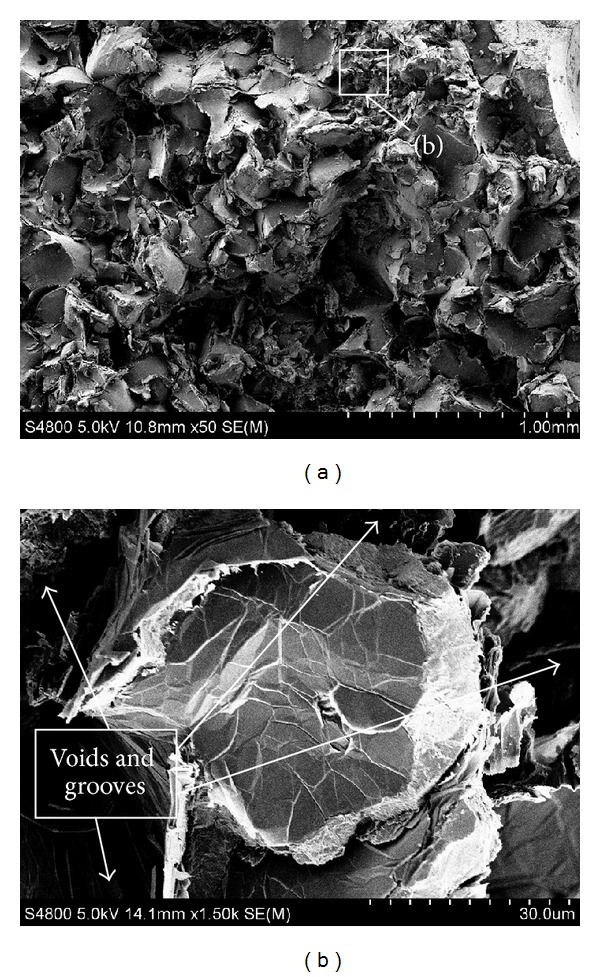
Fracture appearances of HT250 sample: (a) macroappearance SEM × 50, (b) SEM × 1500.

The HT250 substrate fracture appearance is shown in Figure 9(a). The macrofracture mainly contains serration and lamellar fragments. The large cleavage plane is flat and glossy, which belongs to brittle fracture, whereas the small cleavage plane is partly accompanied with lacerated fragments. Considering the plasticity of pearlite and ferrite, part of the appearance presents characteristics of ductile fracture. In addition, the cleavage cracks mainly propagate along thick flaky graphite. Thus, plenty of voids and grooves which are caused by the peeling graphite are found around the fractured substrate, as exhibited in Figure 9(b). The macrobrittle fracture of HT250 substrate contains microductile fracture behavior.

**Figure 10:**
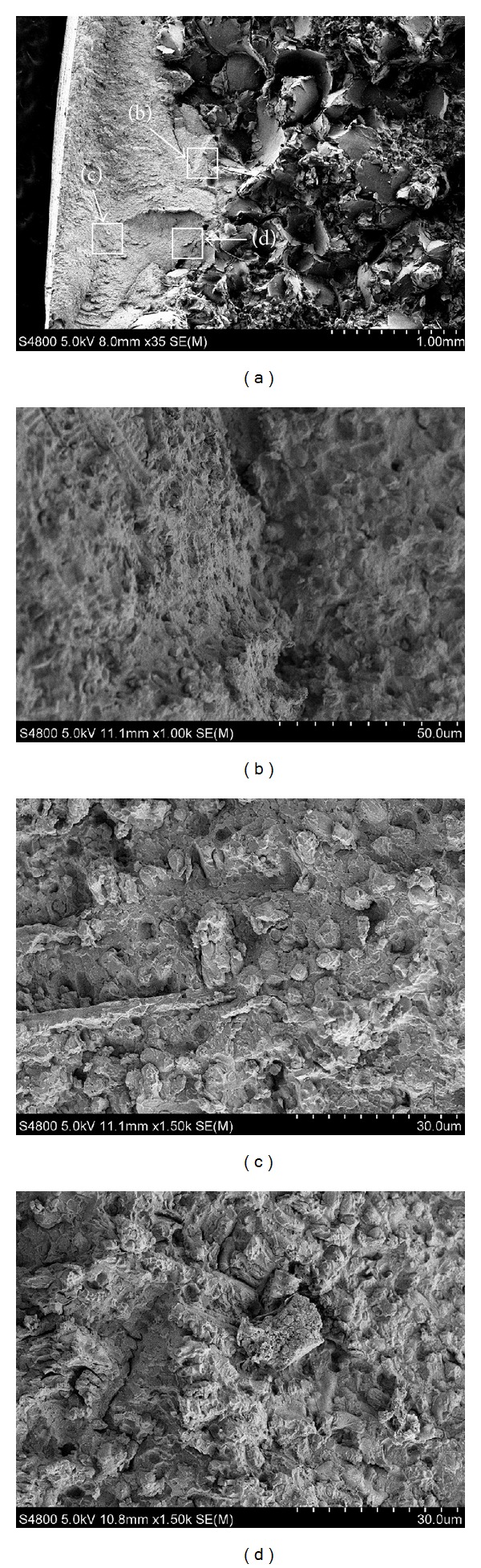
Fracture appearances of Fe314 sample: (a) macroappearance SEM × 35, (b) BZ SEM × 1000, (c) RZ SEM × 1500, and (d) BZ SEM × 1500.

**Figure 11:**
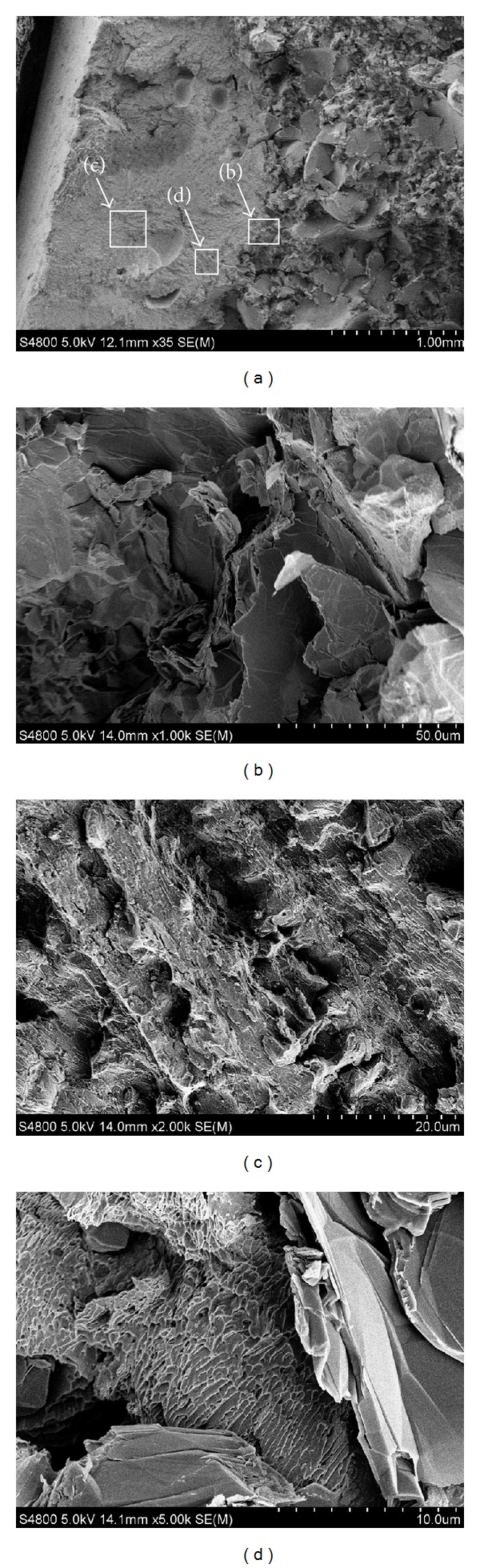
Fracture appearances of 316L sample: (a) marcoappearance SEM × 35, (b) BZ SEM × 1000, (c) RZ SEM × 2000, and (d) BZ SEM × 2000.

The macro- and microfracture morphologies of the RZ in the bending samples of Fe314 and 316L are shown in Figures 10 and 11, respectively. In the vertical direction of laser scanning, the characteristics of the transverse fracture of the RZ and the substrate are completely different. A transition in the bond zone is observed and no obvious delamination is found between the RZ and the substrate, which indicates that the bonding is tight and the fracture consistency is excellent. Part of the macrofracture surface of Fe314 RZ is flat, as shown in Figure 10(a). Fibrous zone and radial fragments can be observed and the cracks propagate along the divergence direction. A large amount of independent cracks interconnect and propagate to produce the fracture, which exhibits the characteristics of ductile fracture.

To investigate the fracture mechanism at different positions, two positions in BZ and RZ are observed with high magnification, respectively, as shown in Figures 10(c) and 10(d). Both fractures are of typical intergranular dimple morphologies. Various columnar and cellular grains are torn and obvious dimple interspace is produced. The microstructure analysis indicates that a small amount of graphite precipitated flaky hard martensite, and lower bainite remains in this area. Deep grooves are induced by the separation of graphite from the fracture. Characteristics of intergranular fracturing are evident in micromorphological analysis. By comparing the dimple morphologies, grains in Figure 10(c) are more tiny and littered than that in Figure 10(d). BZ is located in the remelting area between the double repaired layers, and the grains are coarse and directional, consistent with the microstructure analysis. Therefore, the intergranular dimple fracturing of the microarea is between the ductile and brittle fracturing characteristics, and the macroarea presents mixed fracturing mechanism of ductile and brittle fracturing.

The appearance of the 316L repaired sample fracture is shown in Figure 11. Fracture microstructure of RZ is more compact (Figure 11(a)). The radial fragments of the fracture are not clear and shear lips are observed. The magnification of bond zone in Figure 11(b) illustrates that there is a transition from quasicleavage brittle fracture to ductile fracture from substrate to RZ.  Figure 11(d) is a higher magnification of bond zone morphology, which shows that the fracturing characteristics of diverse parts have evident difference. The right side has a lamellar brittle fracture, whereas the left side is composed of tiny dimple ductile fracture. The micromorphology in RZ shown in Figure 11(c) illustrates a similar intergranular fracture condition with Fe314. However, as the RZ of 316L is austenite stainless steel, microstructure is more compact and precipitation from grain boundary is barely visible. Therefore, this area has good toughness. The tear mark is more obvious at the grain boundaries. Intergranular dimples are deep, and the strengthening effect of grain reticular structure is better. Meanwhile, fracture toughness is closely related to microstructure. The amount of grain boundary increases with grain refinement. Consequently, fracturing paths increase and the crack propagation energy is weakened. Thus, the preventive effect of grain boundary on crack propagation increases and costs more energy to propagate across the grain boundaries.

### 4.3. Load-Displacement Curve of Three-Point Bending Test

**Figure 12:**
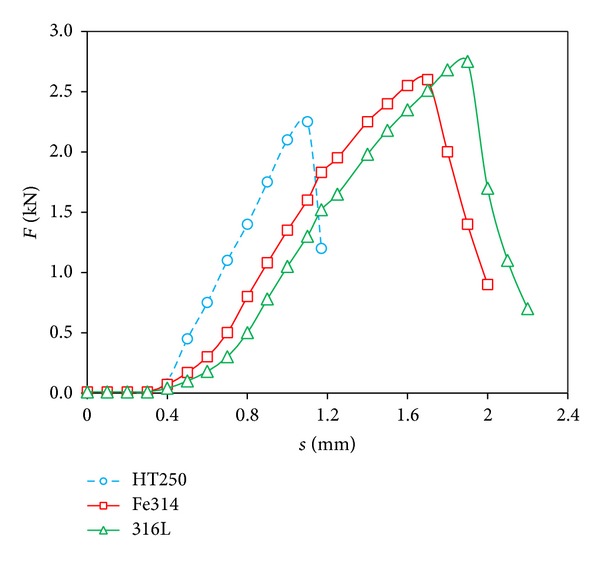
Load-displacement curve.

The load (*F*) and displacement (*s*) curve of fracturing process is illustrated in Figure 12. For the substrate HT250, the load reaches 2.23 kN and then the sample fractures suddenly. The tendency of the *F*-*s* curve is like a straight line, which indicates that the fracture is typical brittle fracture. The crack propagates quickly under large stress and the load declines rapidly from 2.23 kN to 0 kN. For the repaired samples, the displacement under the same load is larger, which proves that the toughness is increased. The peak values of the load of Fe314 and 316L sample are 2.6 kN and 2.75 kN, respectively, which are both higher than that of substrate. This is because the microstructure of RZ is compact and refined, which mainly includes austenite, pearlite, and residual austenite. Besides, there is little flaky graphite in RZ. These contribute the higher tensile strength of repaired samples. The crack is restrained when it propagates to the boundary of RZ and then followed by a slow propagation. As shown in Figure 12, there is a transitory yield procedure around the peak load. When the load is beyond the yield strength, the crack propagates across RZ, and the repaired samples fracture eventually.

### 4.4. Crack Prevention Behavior of the RZ

**Figure 13:**
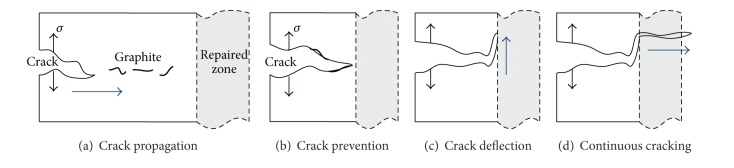
Cracking model of repaired layer.

The main crack remains vertical with maximum tensile stress direction when cast iron substrate fractures. More branches appear at the sides of the main crack and interconnect gradually within the graphite cavity. From the analysis of the fracturing characteristics of samples, laser RZ has positive effect on the fracture prevention when sample is under impact of external concentrated stress. A cracking model is established to depict the fracturing process of the bending test, which is similar to the model for crack propagation process, which is stated in [13]. The cracking model is illustrated in Figure 13 and the arrows present the crack propagation direction.

Crack propagation (Figure 13(a)) is initiated under effect of steady tensile stress at the boundary within one side of sample *i*. The strength of flaky graphite in grey cast iron is extremely low and flaky graphite acts as cavities in substrate. The sharp corners of flaky graphite can cause stress concentration easily, which promote the crack propagation. Therefore, the initiated crack propagates along graphite and substrate between the closest graphite under the external bending stress [13]. This can be verified by the fracture morphology of HT250 substrate in the three-point bending test. The cleavage crack propagates along the thick flaky graphite, as illustrated in Figure 9. With the process of bending test, the flaky graphite along the cracking direction then turns into a crack propagation path, in which the substrate is torn quickly. As the crack propagates closer to the RZ, the graphite amount in BZ declines rapidly, and the microstructure is more compact, as shown in Figure 13(b). The lack of graphite cut off the propagation routine of crack and the compact RZ microstructure with better strength and hardness makes the crack propagation much difficult [13]. With the effect of steady tensile stress, the crack propagation energy accumulates at the tip of crack, which induces the crack wider [21]. Crack propagation along the original direction suffers more resistance, and propagation direction changes from transverse to longitudinal direction. New stress concentration point needs to be found for the crack propagation as exhibited in Figure 13(c). When the crack encounters local abundant graphite or existing microcracks and when the accumulated crack energy reaches a high value, a crack source is generated at the tension side of the RZ. Consequently, the stress concentrates again, and the crack direction reverts to transverse direction as shown in Figure 13(d). To some extent, the RZ has plasticity resistance of tensile stress; the maximum force is a little higher than the substrate without laser repairing. Finally, the crack propagates rapidly in the RZ and leads to sample fracture.

From the analysis of cracking model of the RZ, the characteristic effect of laser RZ is the deflection of cracking direction. Moreover, the effect is clearer when cracking direction is vertical with laser scanning direction. The deflection of crack direction presents the effective function of the material at this area to prevent cracks. Compared with linear cracking, the frequent change of cracking direction prolongs the propagation path and requires more energy to keep the cracking state. Intensity factor of effective stress at crack front end and crack propagation rate declines with decreasing effective stress of cracking. Therefore, the repaired sample exhibits characteristics of good cracking prevention.

## 5. Conclusions

1. Laser repairing of grey cast iron surface is achieved with iron-based Fe314 and 316L alloy powder. The microstructure is compact and homogeneous, and the metallurgical bonding is reliable. Large carbon concentration gradient is generated between the RZ and substrate. Fe314 alloy powder is more suitable for laser repairing of grey cast iron surface.
2. The bottom of Fe314 RZ consists of dendrite with similar growth direction that extends continuously to the secondary RZ. The secondary repairing makes the grains extensive with inheritance. Microstructure near the top surface is composed of equiaxed grains group with improved toughness and machinability of substrate. Microstructure of 316L RZ comprises austenite cellular grain and the grains exhibit characteristic of inherited growth.
3. Laser repairing keeps the original strength of cast iron base material. Base material and RZ exhibited good bonding strength and cracking resistance. The cracking prevention and deflection process is obtained using a cracking model of the RZ. Laser repairing improves the crack resistance and toughness of the overall material.

## Figures and Tables

**Table 1:** Laser repairing processing parameters.

	Laser power (W)	Scan rate (mm/min)	Spot radius (mm)
1st layer	*P*_1_ = 2800 to 3200	*v*_1_ = 360 to 540	*r*_1_ = 3 to 4
2nd layer	*P*_2_ = 2800 to 3200	*v*_2_ = 1.2 × *v*_1_	*r*_2_ = 1.1 × *r*_1_

**Table 2:** Chemical compositions of HT250 (wt.%).

C	Si	Mn	P	S	Fe
3.55	1.58	0.76	0.09	0.08	Bal.

**Table 3:** Chemical composition of the alloy powder used in grey cast iron repair (wt.%).

	C	Si	Cr	B	Mn	Mo	P	S	Ni	Fe
Fe314	0.1	1	15	1	—	—	—	—	10	Bal.
316L	0.03	0.8	16	—	2	2.4	0.03	0.02	12.5	Bal.

## References

[1] Tian W, Liao W, Liu C, Yu L, Tang J (2009). Green remanufacture technology and mode of the key components of railway car. *China Mechanical Engineering*.

[2] Xu BS, Liu SC, Shi PJ (2006). Contribution of remanufacturing engineering and surface engineering to cycle economy. *China Surface Engineering*.

[3] Zhong M, Liu W (2008). Leading areas and hot topics on global laser materials processing research. *Chinese Journal of Lasers*.

[4] Dong L, Yang X, Zhang H, Lei K (2012). Path generation for repairing damaged parts of free-form surfaces in laser remanufacturing. *Chinese Journal of Lasers*.

[5] Ma XD, Lei Y, Liu R (2010). The application of laser cladding technique in dies repairing. *Lubrication Engineering*.

[6] Wang WF, Wang MC, Zhang J, Sun FJ, Huang DW (2008). Research on the microstructure and wear resistance of titanium alloy structural members repaired by laser cladding. *Optics and Lasers in Engineering*.

[7] Capello E, Colombo D, Previtali B (2005). Repairing of sintered tools using laser cladding by wire. *Journal of Materials Processing Technology*.

[8] Kathuria YP (2000). Some aspects of laser surface cladding in the turbine industry. *Surface and Coatings Technology*.

[9] Sridhar K, Katkar VA, Singh PK, Haake JM (2007). Dry sliding friction wear behaviour of high power diode laser hardened steels and cast iron. *Surface Engineering*.

[10] Luan J, Yan M, Zhou Z (2003). Crack resistance and wear resistance of laser clad layer on the surface of cast iron. *Chinese Journal of Materials Research*.

[11] Ocelík V, de Oliveira U, de Boer M, de Hosson JTM (2007). Thick Co-based coating on cast iron by side laser cladding: analysis of processing conditions and coating properties. *Surface and Coatings Technology*.

[12] Tong X, Zhou H, Chen W (2009). Effects of pre-placed coating thickness on thermal fatigue resistance of cast iron with biomimetic non-smooth surface treated by laser alloying. *Optics and Laser Technology*.

[13] Tong X, Zhou H, Ren L, Zhang Z, Zhang W, Cui R (2009). Effects of graphite shape on thermal fatigue resistance of cast iron with biomimetic non-smooth surface. *International Journal of Fatigue*.

[14] Zhao Y, Ren L, Tong X, Zhou H, Chen L (2008). Frictional wear and thermal fatigue behaviors of biomimetic coupling materials for brake drums. *Journal of Bionic Engineering*.

[15] Yi P, Xu P, Yin K, Li C, Liu Y (2013). Laser thermo-repairing process modeling and thermal response analysis on gray cast iron surface. *Chinese Journal of Lasers*.

[16] Yi P, Liu Y, Shi Y, Jang H, Lun G (2011). Effects analysis of ambient conditions on process of laser surface melting. *Optics and Laser Technology*.

[17] Shi Y, Liu Y, Yi P, Hu J (2012). Effect of different heating methods on deformation of metal plate under upsetting mechanism in laser forming. *Optics and Laser Technology*.

[18] Zhong M, Liu W, Ning G, Yang L, Chen Y (2004). Laser direct manufacturing of tungsten nickel collimation component. *Journal of Materials Processing Technology*.

[19] Pinkerton AJ, Li L (2004). Multiple-layer cladding of stainless steel using a high-powered diode laser: an experimental investigation of the process characteristics and material properties. *Thin Solid Films*.

[20] Shi JP, Han D, Tang AM (2009). The fracture analysis of ductile iron fracture test under complex stress condition. *Journal of Xi'an University of Technology*.

[21] Zhou H, Tong X, Zhang Z, Li X, Ren L (2006). The thermal fatigue resistance of cast iron with biomimetic non-smooth surface processed by laser with different parameters. *Materials Science and Engineering A*.

